# The Lyme disease spirochete *Borrelia burgdorferi* induces inflammation and apoptosis in cells from dorsal root ganglia

**DOI:** 10.1186/1742-2094-10-88

**Published:** 2013-07-18

**Authors:** Geeta Ramesh, Lenay Santana-Gould, Fiona M Inglis, John D England, Mario T Philipp

**Affiliations:** 1 Division of Bacteriology and Parasitology, Tulane National Primate Research Center, Covington, LA, USA; 2 Department of Cell and Molecular Biology, Tulane University, New Orleans, LA, USA; 3 Department of Neurology, Louisiana State University Health Sciences Center, New Orleans, LA, USA

**Keywords:** Lyme neuroborreliosis, *Borrelia burgdorferi*, Dorsal root ganglia, Schwann cells, CCL2/MCP-1, IL-6, IL-8, Neuronal apoptosis, Dexamethasone

## Abstract

**Background:**

Lyme neuroborreliosis (LNB), caused by the spirochete *Borrelia burgdorferi*, affects both the peripheral and the central nervous systems. Radiculitis or nerve root inflammation, which can cause pain, sensory loss, and weakness, is the most common manifestation of peripheral LNB in humans. We previously reported that rhesus monkeys infected with *B*. *burgdorferi* develop radiculitis as well as inflammation in the dorsal root ganglia (DRG), with elevated levels of neuronal and satellite glial cell apoptosis in the DRG. We hypothesized that *B*. *burgdorferi* induces inflammatory mediators in glial and neuronal cells and that this inflammatory milieu precipitates glial and neuronal apoptosis.

**Methods:**

To model peripheral neuropathy in LNB we incubated normal rhesus DRG tissue explants with live *B*. *burgdorferi ex vivo* and identified immune mediators, producer cells, and verified the presence of *B*. *burgdorferi* in tissue sections by immunofluorescence staining and confocal microscopy. We also set up primary cultures of DRG cells from normal adult rhesus macaques and incubated the cultures with live *B*. *burgdorferi*. Culture supernatants were subjected to multiplex ELISA to detect immune mediators, while the cells were evaluated for apoptosis by the *in situ* TUNEL assay. A role for inflammation in mediating apoptosis was assessed by evaluating the above phenomena in the presence and absence of various concentrations of the anti-inflammatory drug dexamethasone. As Schwann cells ensheath the dorsal roots of the DRG, we evaluated the potential of live *B*. *burgdorferi* to induce inflammatory mediators in human Schwann cell (HSC) cultures.

**Results:**

Rhesus DRG tissue explants exposed to live *B*. *burgdorferi* showed localization of CCL2 and IL-6 in sensory neurons, satellite glial cells and Schwann cells while IL-8 was seen in satellite glial cells and Schwann cells. Live *B*. *burgdorferi* induced elevated levels of IL-6, IL-8 and CCL2 in HSC and DRG cultures and apoptosis of sensory neurons. Dexamethasone reduced the levels of immune mediators and neuronal apoptosis in a dose dependent manner.

**Conclusion:**

In this model, *B*. *burgdorferi* induced an inflammatory response and neuronal apoptosis of DRG. These pathophysiological processes could contribute to peripheral neuropathy in LNB.

## Background

Lyme neuroborreliosis (LNB), the form of Lyme disease that affects the nervous system, is manifest in about 15% of patients. Both the central and peripheral nervous systems may be affected [1-5]. Patients with central nervous system (CNS) involvement may complain of headache, flu-like symptoms, fatigue, memory loss, learning disability or depression. Infection of the peripheral nervous system (PNS) with *Borrelia burgdorferi*, the Lyme disease bacterium, may result in facial nerve palsy, pain, sensory loss, or muscle weakness.

Clinically, LNB may manifest as meningitis, typically characterized by lymphocytic pleocytosis in the cerebrospinal fluid (CSF), meningoradiculitis (also known as Bannwarth’s syndrome), cranial neuritis, encephalopathy, peripheral neuropathy and, less commonly, encephalitis and encephalomyelitis. Radiculitis, or inflammation in the dorsal roots, is the most common manifestation of untreated LNB in humans [2,4]. LNB patients may also experience a wide array of neurological symptoms as a result of white matter inflammation in the brain and spinal cord that results in a sub-acute multiple sclerosis (MS)-like manifestation [6,7].

Pathology reports from human cases of LNB have described lymphocyte and plasma cell infiltration in the meninges and perivascularly in the nerve roots, dorsal root ganglia (DRG), as well as demyelination in the brain and spinal cord [8-13]. Typically, peripheral nervous system Lyme disease is associated with patchy multifocal axonal loss with epineural perivascular inflammatory infiltrates or perineuritis [14-18].

In an earlier study, histopathological evaluation showed varying degrees of necrosis in the sensory ganglia of rhesus macaques that were infected with *B*. *burgdorferi*, in addition to positive immunostaining with monoclonal antibodies against a 7.5 kDa lipoprotein of *B*. *burgdorferi*[19]. In an *in vivo* experiment in which we inoculated *B*. *burgdorferi* into the cisterna magna of rhesus macaques, analysis of the CSF within one-week post-inoculation showed increased levels of IL-6, IL-8, CCL2, and CXCL13, accompanied by a monocytic/lymphocytic pleocytosis. This inflammatory response was concomitant with histopathological changes consistent with acute neurologic Lyme disease, such as leptomeningitis and radiculitis. In addition, we observed elevated levels of neuronal and satellite glial cell apoptosis in the DRG of infected animals as compared to uninfected controls and documented the presence of IL-6 in DRG neurons of infected animals [20].

The mechanisms underlying the pathogenesis of peripheral LNB are not clearly understood. Based on our observations, we hypothesized that *B*. *burgdorferi* was able to induce inflammatory mediators in glial and neuronal cells and that this inflammatory context precipitated glial and neuronal apoptosis. As a model to study the mechanisms underlying peripheral neuropathy seen in patients with Lyme neuroborreliosis, we obtained fresh rhesus DRG tissue explants and allowed live Lyme disease bacteria to interact with the tissue explants *ex vivo* to allow for accumulation of intracytoplasmic proteins. Cryo-sections were stained to detect immune mediators, the phenotypes of producer cells and the presence of *B*. *burgdorferi* spirochetes, and were visualized using confocal microscopy. We also set up primary cultures of dorsal root ganglia cells from normal adult rhesus macaques and characterized the cells phenotypically. We then incubated the DRG cultures with live *B*. *burgdorferi*. Culture supernatants were used for the detection of immune mediators while the cells were assessed for apoptosis. To determine if inflammation had a role in apoptosis, the above phenomena were evaluated in the presence and absence of various concentrations of the anti-inflammatory drug dexamethasone. Since DRG also contain Schwann cells, which ensheath the dorsal roots, we investigated whether *B*. *burgdorferi* had the potential to induce inflammation in human Schwann cells. The results of these experiments are described below.

## Methods

### Growth and preparation of live spirochetes

*B*. *burgdorferi* B31 clone 5A19 spirochetes, passage 1 to 3 were grown to late logarithmic phase under microaerophilic conditions in Barbour Stoenner-Kelly (BSK) medium, supplemented with 6% rabbit serum (Sigma, St. Louis, MO, USA) and antibiotics (rifampicin at 45.4 mg/mL, fosfomycin at 193 mg/mL and amphotericin at 0.25 mg/mL). Spirochetes were pelleted at 2000 × g for 30 minutes at room temperature. At the end of the run the rotor was left to coast without breaking so as to minimize damage to the live spirochetes. The culture was washed using sterile phosphate buffered saline (PBS) and resuspended in the working medium at the desired density.

### Incubation of dorsal root ganglia explant slices with live spirochetes

DRG tissue was obtained immediately after euthanasia from three normal rhesus macaques and placed in PBS pH 7.2 (Invitrogen, Grand Island, NY, USA) at room temperature. The tissue was sliced using sterile number 21 scalpels (Personna Medical, Verona, VA, USA). The slices were placed in separate wells of 12-well plates (Fisher Scientific, Fair Lawn, NJ, USA), each containing 2 ml of RPMI 1640 medium (Invitrogen) supplemented with 10% fetal bovine serum (FBS) (Invitrogen). Live *B*. *burgdorferi* spirochetes at a final density of 1 × 10^7^/mL were added to some wells. Some wells received, in addition, brefeldin A (Molecular Probes, Eugene, OR, USA), a fungal metabolite that blocks protein transport [21] at a final concentration of 10 μg/mL. Corresponding control slices were also held in medium plus brefeldin A without spirochetes. The DRG slices were then incubated at 37°C for four hours in a humidified 5%-CO_2_ incubator. At the end of the four-hour incubation, tissue slices were fixed in 2% paraformaldehyde in PBS pH 7.0 (USB, Cleveland, OH, USA) and cryopreserved as described earlier [22].

### Immunofluorescence staining for detection of intracytoplasmic immune mediators

For *in situ* analysis of intracytoplasmic proteins, frozen tissue blocks were cryosectioned into 16-μm sections as previously described [22]. A total of ten cryosections was evaluated per tissue block from each of the above three animals for detection of intracytoplasmic immune mediators. DRG tissue slices were subjected to immunofluorescence staining as previously described [23]. The primary antibodies against various phenotypic markers of cells used were anti-human neurotrophin receptor p75 (p75NTR) polyclonal rabbit antibody presented in rabbit serum used at 1:10 dilution (Millipore, Billerica, MA, USA), anti-human 2′,3′-cyclic nucleotide 3′-phosphodiesterase (CNPase), clone 11-5B mouse IgG1 (Millipore) at 10 μg/mL, anti-human S-100 (Sigma) at 1:500, anti-human neuronal protein NeuN, MAB 377 clone A60, mouse IgG1 (Millipore) at 1:10, or anti-human glial fibrillary acidic protein (GFAP), at 1:200, clone G-A-5 purified mouse immunoglobulin conjugated to Cy3 (Sigma). Primary antibodies for immune mediators were either anti-human IL-6, mouse IgG2a at 1:1000 (ProSpec, Ness Ziona, Israel), anti-human CCL2, mouse IgG1 (5 J): SC-32771 at 1:50 (Santa Cruz Biotechnology, Santa Cruz, CA, USA) or rabbit polyclonal IgG clone ab7814 at 1:50 (AbCam, Cambridge, MA, USA), anti-human IL-8 polyclonal rabbit IgG at 10 μg/mL (RDI, Flanders, NJ, USA). *B*. *burgdorferi* was stained with a polyclonal rabbit antibody against whole *Borrelia* at 1:200 (Accurate Chemicals, Westbury, NY, USA) in combination with a Zenon kit Alexa 647 (Invitrogen). Isotype controls (Sigma) at the concentrations of the respective primary monoclonal antibodies and universal rabbit negative control (Dako Cytomation, Carpinteria, CA, USA) for rabbit polyclonals were also included. All primary antibodies and isotype controls at the appropriate concentrations were prepared in PBS containing 10% normal goat serum (NGS, Invitrogen), 0.2% fish skin gelatin (FSG, Sigma) and 0.02% sodium azide (Sigma), and left on the slides for one hour at room temperature in a humidifying chamber. The slides were then washed with PBS-FT buffer (PBS pH 7.4 containing 0.2% FSG and 0.02% Triton X-100 (MP Biomedicals, Solon, OH, USA) and then held in this buffer for five minutes followed by a rinse with PBS-F buffer (phosphate-buffered saline containing 0.2% FSG).

The relevant secondary antibodies, goat anti-rabbit conjugated to one of the Alexa fluorochromes, Alexa 488 (green), 568 (red) or 633 (blue) (Invitrogen), at a dilution of 1:1000 in PBS containing 10% NGS, 0.2% FSG and 0.02% sodium azide were applied to the tissues and left in the humidified dark slide chamber at room temperature for 30 to 45 minutes. In some cases the Zenon Rabbit IgG labeling kit was used (Invitrogen). Slides were washed with PBS-FT buffer and rinsed with PBS-F buffer as described above and then mounted in anti-quenching medium (Sigma). The stained and mounted slides were stored in the dark at 4°C until they were viewed.

### Rhesus primary dorsal root ganglia cultures

Chamber slides (two wells) with detachable culture slides that were previously coated with poly-D lysine (BD Biosciences, Franklin Lakes, NJ, USA) were coated with mouse laminin (Invitrogen) at a final concentration of 10 μg/mL for a minimum of two hours before seeding the cells. Just before plating the DRG cells, the laminin was removed and wells were rinsed twice with sterile deionized water. Cells were seeded immediately after removal of the laminin. DRG from two adult rhesus monkeys were obtained at necropsy and transported immediately on ice in high glucose (D)MEM F12 (Invitrogen) containing penicillin and streptomycin (P/S) (1 × of 10,000 units of penicillin and 10,000 μg/mL of streptomycin) (Invitrogen) to the biosafety chamber. The DRG were transferred to a petri dish containing Hanks balanced salt solution (HBSS) (Invitrogen). The nerve trunks were cut away using sterile number 21 scalpel blades (Personna Medical). The tissue from about four to six DRG from each animal was minced and transferred to a tube containing 5 mL of 0.25% trypsin ethylenediaminetetraacetic acid (EDTA) (Invitrogen) with 1,000 units of DNAse (Sigma). The minced tissue was transferred to a water bath set at 37°C for 10 to 15 minutes with intermittent shaking. The trypsinization was stopped by adding 25 mL of complete (D)MEM medium containing 10% FBS (Fisher Scientific) and P/S. The complete cell suspension was centrifuged at 1800 to 2000 rpm (300 to 350 g) for ten minutes at room temperature (18°C). The cell pellet was transferred to a clean tube containing 5 mL of complete (D)MEM F12, supplemented with NGF-7S (Invitrogen) at a concentration of 50 ng/mL. (D)MEM was supplemented with fresh L-glutamine at a final concentration of 2 mM. The cells were counted using a hemocytometer and resuspended to a final count of 2 × 10^5^/mL. Cells were seeded at a count of 1 × 10^5^ cells per well, by seeding 500 μl of the above suspension per well. Chambers were left in the humidified CO_2_ incubator for one hour, after which a volume of 1.5 mL of complete (D)MEM F12 medium containing 10% FBS and P/S supplemented with fresh L-glutamine (2 mM), and NGF (50 ng/mL) was added gently to each well drop by drop and left to incubate. Cultures were maintained for a period of six to seven days with medium changes every three to four days. The DRG culture protocol was adapted from previously published protocols for the isolation of DRG neurons from embryonic or adult rats, [24].

### Human Schwann cell cultures

Cryopreserved human Schwann cell (HSC) cultures obtained from ScienCell Inc., (Carlsbad, CA, USA), (isolated from adult human spinal nerves) were revived in tissue culture flasks coated with poly-L-lysine as per the manufacturer’s instructions and maintained in the supplied Schwann cell medium (SCM) consisting of basal medium, 5% FBS, 1% Schwann cell growth supplement and 1 x antibiotic mixture of penicillin/streptomycin. Several aliquots of the above-revived culture (passage two) were frozen back as per the manufacturer’s instructions and thawed and revived as needed for experiments. Cultures that were 80% confluent were trypsinized and re-seeded onto poly-L-lysine chamber slides containing two wells at a seeding density of 1 × 10^4^ cells per well, as recommended by ScienCell Inc., after which they were maintained in SCM for three days prior to commencing experiments. Extra wells were seeded to evaluate final cell count prior to incubation with live *B*. *burgdorferi* at the desired multiplicity of infection (MOI). Duplicate wells were seeded for each condition for each of the two experiments, set up using different passage-two stocks. Both basal media and Schwann cell supplement were supplied by the manufacturer and their composition is proprietary. HSC maintained on poly-L-lysine-coated chamber slides were used for the evaluation of phenotypic markers and secreted immune mediators using a Human 23-plex Cytokine-Chemokine Array kit (Millipore) described below.

### Immunofluorescence staining and confocal microscopy for detection of the expression of Schwann cell, neuronal and satellite glial cell markers

Medium was removed from HSC cultures or DRG cultures and cells were fixed in 2% paraformaldehyde (PFA) followed by post-fixation permeabilization using a mixture of ethanol:acetic acid (2:1) (Sigma) for five minutes at −20°C. The slides were then detached from the chamber and processed for immunofluorescence staining as previously described [25].

Schwann cell cultures were evaluated for the expression of myelin basic protein (MBP), using rabbit polyclonal anti-human MBP Clone AB 980 at 1:100 (Millipore, Billerica, MA, USA), and CD-90 using mouse monoclonal IgG1 anti-human THY-1 (CD90) at 10 μg/mL (Millipore), in addition to CNPase, p75 NTR and S-100, while DRG cultures were evaluated for the expression of NeuN, GFAP, S-100, CNPase and p75 NTR, as described above in the section entitled ‘Immunofluorescence staining for detection of intracytoplasmic immune mediators’.

Confocal microscopy was performed using a Leica TCS SP2 confocal microscope equipped with three lasers (Leica Microsystems, Exton, PA, USA). Images of individual channels were merged to obtain images containing all channels. Photoshop CS3 (Adobe Systems Inc., San Jose, CA, USA) was used to assign colors to each fluorochrome.

### Evaluation of the role of inflammation in mediating neuronal apoptosis in dorsal root ganglia cultures using the anti-inflammatory drug dexamethasone

DRG cell cultures were seeded as described above in chamber slides for evaluation of apoptosis or for evaluation of immune mediators and maintained in growth medium for six days. Prior to stimulation with live *B*. *burgdorferi*, DRG cultures were incubated with various concentrations of dexamethasone (water soluble), 5 μM, 15 μM and 150 μM (Sigma) for 24 hours at 37°C, after which they were washed and then incubated in fresh growth medium containing the respective concentrations of dexamethasone and live *B*. *burgdorferi* at a MOI of 10:1 at 37°C for 24 hours and devoid of P/S. Similar concentrations of dexamethasone as those mentioned above have been reported to inhibit the production of CCL2 in mice microglia [26]. The dexamethasone that we used in our experiments is supplied as a water-soluble formulation consisting of dexamethasone and a carrier substance (2-hydroxypropyl)-β-cyclodextrin (HPC). The effect of HPC alone, at respective molar concentrations found in the concentrations of dexamethasone that we used in our experiments, was done by incubating DRG cultures as described above in the presence and absence of *B*. *burgdorferi* and the above mentioned concentrations of carrier alone at 15 μM, 45 μM and 450 μM, respectively.

After 24 hours, culture supernatants were collected and processed for quantification of inflammatory mediators, and cells were fixed and evaluated for apoptosis by the *in situ* terminal deoxynucleotidyl transferase mediated UTP nick end labeling (TUNEL) assay as described below. Medium controls that were pretreated and then incubated with the same respective concentrations of dexamethasone but without the addition of live *B*. *burgdorferi* were also included.

### Evaluation of apoptosis by *in situ* TUNEL assay

Cells contained in chamber slides were labeled for NeuN by immunofluorescence staining as described above. Slides were then fixed with 2% PFA, washed three times with PBS by rinsing the slides in PBS and holding them in PBS for two minutes between washes. Slides were then subjected to the TUNEL-ApopTagPlus fluorescein *in situ* apoptosis assay (Chemicon, Temecula, CA, USA) as per the manufacturer’s instructions. Slides were then mounted as described above and stored at 4°C in the dark until viewed. The percentage of apoptotic neurons from ten fields was evaluated from each chamber area by counting the total number of NeuN-positive cells (at least 500 cells) from each of the chamber areas, followed by the percentage of cells that showed co-localization of both the TUNEL signal and NeuN expression. All counts were made by viewing slides under a fixed magnification of 63X (corresponding to an area of 0.05 mm^2^) using the confocal microscope. Cultures were also stained with S-100 prior to doing the TUNEL assay as described above to detect apoptotic satellite glial cells.

### Stimulation of human Schwann cell cultures with live *B*. *burgdorferi* for evaluation of immune mediators

*B*. *burgdorferi* strain B31 5A19 passage 3 was prepared as described above. The HSC cultures were washed in SCM devoid of P/S. The *B*. *burgdorferi* culture was resuspended in SCM devoid of P/S, at the desired MOI. Controls with no spirochetes were also included. Cultures were incubated for 48 hours in a humidified 5% CO_2_ incubator set at 37°C. At the 48-hour time point culture supernatants were collected for evaluation of inflammatory mediators. Culture supernatants were centrifuged at 4°C at 2000 × g to remove any suspended bacteria and the supernatant was aliquoted and stored at −70°C until used.

### Evaluation of immune mediators from culture supernatants

The concentrations of cytokines and chemokines present in the culture supernatants from rhesus DRG were quantified using the MILLIPLEX MAP Non-Human Primate Cytokine Magnetic Bead Panel - Premixed 23 Plex, PCYTMG-40 K-PX23 Cytokine-Chemokine Array kit (Millipore) following the manufacturer’s instructions. The analytes detected by this panel are: G-CSF, GM-CSF, IFN-γ, IL-10, IL-12/23 (p40), IL-13, IL-15, IL-17, IL-18, IL-1ra, IL-1β, IL-2, IL-4, IL-5, IL-6, IL-8, CCL2, CCL3, CCL4, TGF-α, TNF-α, VEGF and sCD40L. The concentrations of cytokines and chemokines present in the culture supernatants from HSC cultures described above were quantified using the MILLIPLEX Human Cytokine/Chemokine - Premixed 14 Plex, MPXHCYTO60KPMX14 Cytokine-Chemokine Array kit (Millipore) following the manufacturer’s instructions. The analytes detected by this panel are: GM-CSF, IFN-γ, IL-10, IL-12 (p70), IL-13, IL-1β, IL-2, IL-4, IL-5, IL-6, IL-7, IL-8, CCL2 and TNF-α. The multiplex plate was read using a Bio-Plex 200 Suspension Array Luminex System (Bio-Rad, Hercules, CA, USA).

### Statistical evaluation

The unpaired-two tailed t test was used to evaluate the statistical significance between means of data sets, using Graphpad Prizm software (Graph Pad Software Inc.) version 4. A *P* value of 0.05 or lower was considered to be statistically significant.

## Results

### Visualization of CCL2, IL-6 and IL-8 in cells from dorsal root ganglia tissue explants after *ex vivo* stimulation with live spirochetes

**Figure 1:**
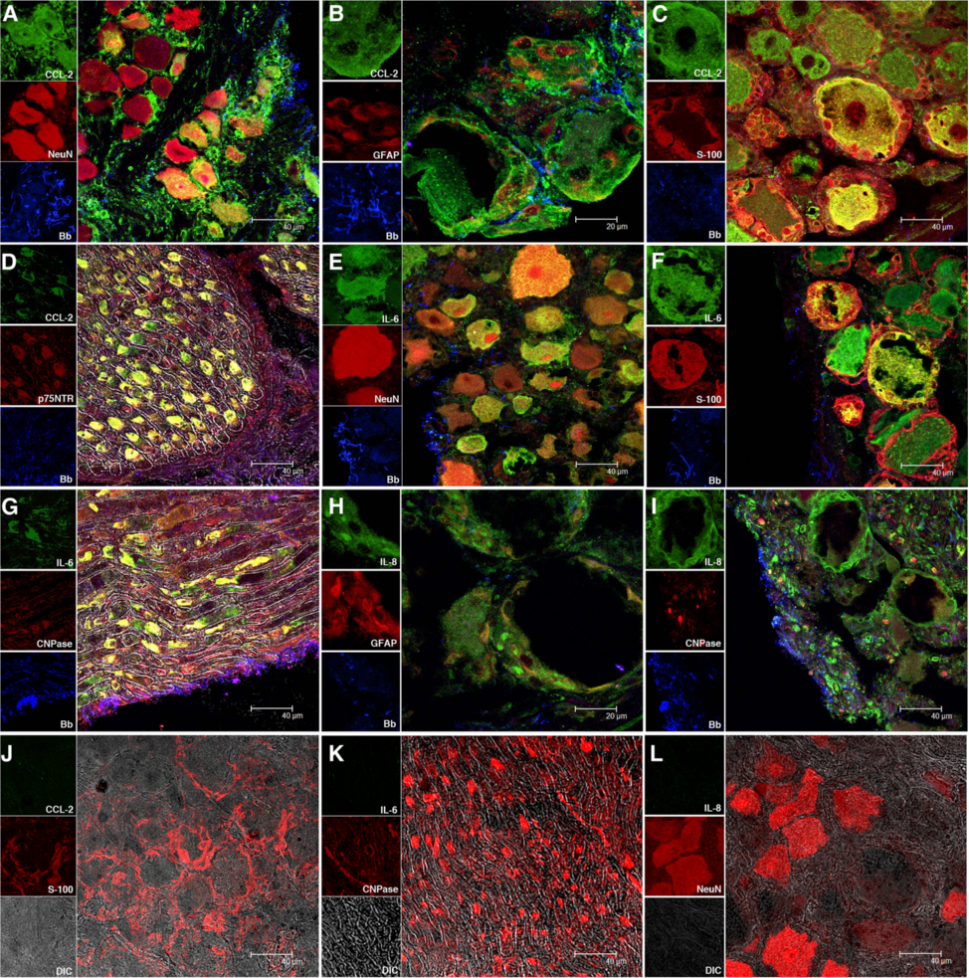
**Visualization of CCL2, IL-6, IL-8 and *B*. *burgdorferi*. (A)** CCL-2 (green) inside neurons stained red with NeuN. The signal for CCL-2 is also in satellite glia and dorsal root nerves. **(B)** CCL-2 in green in satellite glia stained red with GFAP-cy3. **(C)** CCL-2 in green in satellite glial and neurons stained red with S-100. CCL-2 also co-localizes with Schwann cells staining positive with p75NTR, in red in the dorsal root **(D)**. Unstained nerve tissue is gray as imaged by differential interference contrast (DIC). The spirochetes in A-D appear blue stained with antibody against whole *B*. *burgdorferi*, in combination with a Zenon labeling Kit Alexa 647. **(E)** IL-6 (green) localized inside neurons staining red with NeuN. The signal for IL-6 is also in areas occupied by satellite glia. **(F)** IL-6 in green within the satellite glia and neurons stained red by S-100. The signal for IL-6 is also seen in areas occupied by sensory neurons. **(G)** IL-6 (green) in Schwann cells stained with p75NTR, in red in the dorsal root; the remaining unstained nerve tissue is gray as imaged by DIC. The spirochetes appear blue in E-G. **(H)** IL-8 (green) inside satellite glia stained positive for GFAP (red). The signal for IL-8 is not seen in areas occupied by neurons. **(I)** IL-8 (green) co-localized within Schwann cells stained with CNPase (red) in the dorsal root. Spirochetes appear blue in **H** and **I**. **(J**, **K** and **L)** show the absence of CCL2 , IL-6 and IL-8, respectively, in control sections (green signal) that were also stained in parallel for S-100, CNPase or NeuN (all appearing red). The unstained tissue appears gray as imaged by DIC. Results indicate representative images of DRG explants from three adult rhesus macaques. CNPase, 2′,3′-cyclic nucleotide 3′-phosphodiesterase; DRG, dorsal root ganglia; GFAP, glial fibrillary acid protein.

The DRG tissue explants stimulated *ex vivo* with spirochetes in the presence of brefeldin A showed localization of CCL-2 in sensory neurons (Figure 1A). CCL-2, which is stained green in this tissue, is localized inside the neurons giving a yellow signal due to the co-localization of red and green within the neurons. CCL-2 is also seen in areas occupied by satellite glial cells and dorsal root in the DRG. CCL-2 in green is seen co-localizing with the satellite glial cell marker GFAP (Figure 1B). Both satellite glial cells and sensory neurons in the DRG that are known to stain positive for S-100 [27], seen in red, show the presence of CCL-2 (Figure 1C). CCL-2 is also seen to co-localize with Schwann cells in the dorsal root, staining positive with p75NTR, seen in red (Figure 1D), while remaining unstained nerve tissue is seen as gray. The spirochetes appear blue in Figure 1A-1D.

Similarly, the cytokine IL-6 was seen to be localized in sensory neurons (Figure 1E and Figure 1F, respectively) and in satellite glial cells (Figure 1F). Schwann cells also showed the presence of IL-6 (Figure 1G).

The chemokine IL-8 was localized inside satellite glial cells (Figure 1H) and in Schwann cells (Figure 1I) but not in neurons. The lack of IL-8 production in neurons was confirmed by staining the section for detection of IL-8 and with NeuN, a specific marker for neurons (not shown). CCL2, IL-6 and IL-8 were not detected in control sections of DRG explants that were held in medium containing brefeldin A for four hours in the absence of *B*. *burgdorferi*. Representative images showing the absence of CCL-2, IL-6 and IL-8 (green signal) in control sections (no *B*. *burgdorferi*) that were also stained in parallel for S-100, CNPase or NeuN (all appearing red) are shown in Figure 1J, Figure 1K, and Figure 1L, respectively. The unstained tissue appears gray. No signal was detected in sections stained with isotype controls for respective primary antibodies followed by corresponding secondary antibodies for the mediators or the cell markers (not shown).

### Phenotypic cell markers expressed in primary cultures of dorsal root ganglia cells

**Figure 2:**
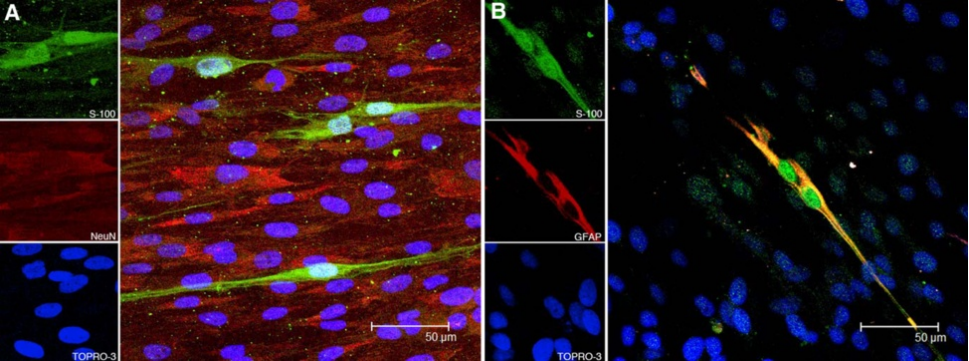
**Expression of the neuronal marker NeuN and satellite glial cell markers GFAP and S-100 in primary rhesus DRG cultures.** Primary rhesus DRG cultures showing a mat of sensory neurons staining positive for the neuronal marker NeuN (red) **(A)** and a few stray satellite glial cells staining positive for the glial cell markers S-100 and GFAP **(B)**. The nuclei of all cells in A and B appear blue due to staining with the nuclear stain TOPRO3. DRG, dorsal root ganglia; GFAP, glial fibrillary acid protein.

In order to permit a more detailed evaluation of the ability of *B*. *burgdorferi* spirochetes to elicit inflammation and apoptosis in DRG cells, we set up *in vitro* cultures of these cells and characterized their phenotypes. The primary rhesus DRG cultures consisted primarily of a mat of sensory neurons that stained positive for the neuronal marker NeuN and a few stray satellite glial cells that stained positive for the glial cell marker S-100 (Figure 2A). The satellite glial cells also showed the expression of GFAP (Figure 2B). The sensory neurons in the DRG cultures did not stain positive for S-100, contrary to what we had observed in sensory neurons within DRG tissue explants (Figure 1C and Figure 1F). The nuclei of all cells in Figure 2A and 2B appeared blue due to staining with the nuclear stain TOPRO3. DRG culture slides that were incubated with respective isotype controls of primary antibodies and corresponding secondary antibodies did not show any detectable signal (not shown). DRG cultures did not show specific staining with antibodies to the Schwann cell markers CNPase or p75NTR.

### Expression of MBP, CNPase and p75NTR in human Schwann cell cultures

**Figure 3:**
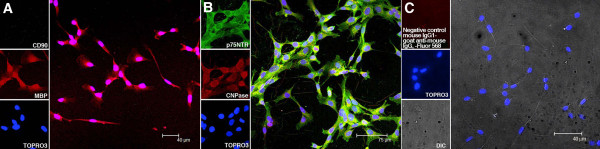
**Expression of MBP, CNPase and p75NTR in HSC cultures. (A)** shows HSC cultures staining with MBP (red) but not CD-90. **(B)** shows the expression of the myelinating marker CNPase (red) and the Schwann cell marker p75NTR (green) in HSC cultures. A representative image of HSC cultures stained with isotype control for mouse IgG1 followed by goat anti-mouse Alexa Fluor 568 is shown in **(C)**. The nuclei of cells appear blue in **A**, **B** and **C** due to staining with nuclear stain TOPRO3. CNPase, 2′,3′-cyclic nucleotide 3′-phosphodiesterase; HSC, human Schwann cell; MBP, myelin basic protein.

As we were not able to detect the presence of Schwann cells in the DRG cultures described above, we resorted to cultivating commercially available HSCs and confirmed their phenotype and purity. HSC cultures showed specific staining with MBP and did not show the expression of CD-90 (Figure 3A). Our HSC cultures also showed the expression of the myelinating marker CNPase and the Schwann cell marker p75NTR (Figure 3B). The HSC cultures did not show any signal when stained with isotype controls for the various primary antibodies at the relevant concentrations followed by secondary antibodies. A representative image of HSC cultures stained with isotype control for mouse IgG1 followed by goat anti-mouse Alexa Fluor 568 is shown in Figure 3C. The nuclei of cells appear blue in Figure 3A, 3B and 3C due to staining with the nuclear stain TOPRO3. The HSC expressed the markers known to be present on adult HSCs, such as MBP [28], CNPase [29] and p75 NTR [30] but did not show the expression of CD90/THY-1, a phenotypic marker of perineural fibroblasts [31], thus signifying the purity of the HSC culture.

### Effect of dexamethasone on the pro-inflammatory response elicited by *B*. *burgdorferi* in primary rhesus dorsal root ganglia cultures

**Figure 4:**
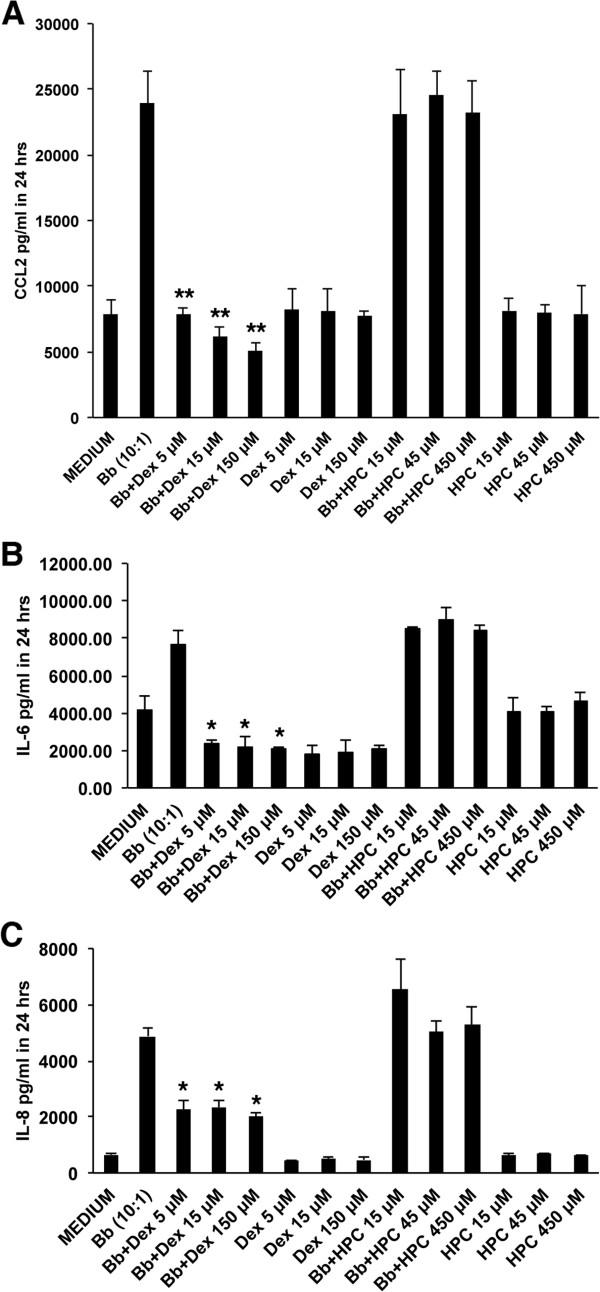
**Effect of dexamethasone on the pro**-**inflammatory response elicited by *B***. ***burgdorferi *in primary rhesus DRG cultures.** The levels of CCL2 **(A)**, IL-6 **(B)** and IL-8 **(C)**, respectively, in rhesus DRG cultures stimulated with live *B*. *burgdorferi* spirochetes for 24 hours at a MOI of 10:1 and in medium controls (* *P* <0.05, ** *P* <0.01) in the presence and absence of dexamethasone and carrier HPC are shown. Data represent mean values and standard deviations between values of duplicate wells that were seeded from DRG cells that were isolated from two adult rhesus macaques. DRG, dorsal root ganglia; HPC, (2-hydroxypropyl)-β-cyclodextrin; MOI, multiplicity of infection.

Live *B*. *burgdorferi* spirochetes incubated with rhesus DRG cultures for 24 hours at a MOI of 10:1 induced significantly elevated levels of CCL2 (Figure 4A), IL-6 (Figure 4B) and IL-8 (Figure 4C) compared to the levels induced in medium controls. The concentration of CCL2 surpassed 20,000 pg/mL, whereas the constitutive level of this chemokine that was produced in medium alone was around 8,000 pg/mL (Figure 4A). The basal concentration of IL-6 was 4,000 pg/mL but reached almost 8,000 pg/mL (Figure 4B) and the basal level of IL-8 production in DRG cultures was around 800 pg/mL but reached about 5,000 pg/mL when stimulated with live *B*. *burgdorferi* for 24 hours (Figure 4C).

Dexamethasone significantly reduced the levels of CCL2 (*P* <0.01), IL-6 (*P* <0.05) and IL-8 (*P* <0.05) as induced by live *B*. *burgdorferi* (MOI of 10:1) in DRG cultures after 24 hours in a dose dependent manner as shown in Figures 4A, 4B and 4C, respectively. We confirmed that the anti-inflammatory effect of the dexamethasone formulation was due to its dexamethasone fraction and not due to the carrier substance (HPC), as HPC alone at 15 μM, 45 μM and 450 μM present in the dexamethasone solutions at the concentrations used above was unable to reduce the levels of *B*. *burgdorferi*-induced immune mediators (Figure 4A, 4B and 4C, respectively). Data represent mean values and standard deviations between values of two independent experiments.

### Inhibiting effect of dexamethasone on apoptosis induced by *B*. *burgdorferi* in primary rhesus dorsal root ganglia cultures

**Figure 5:**
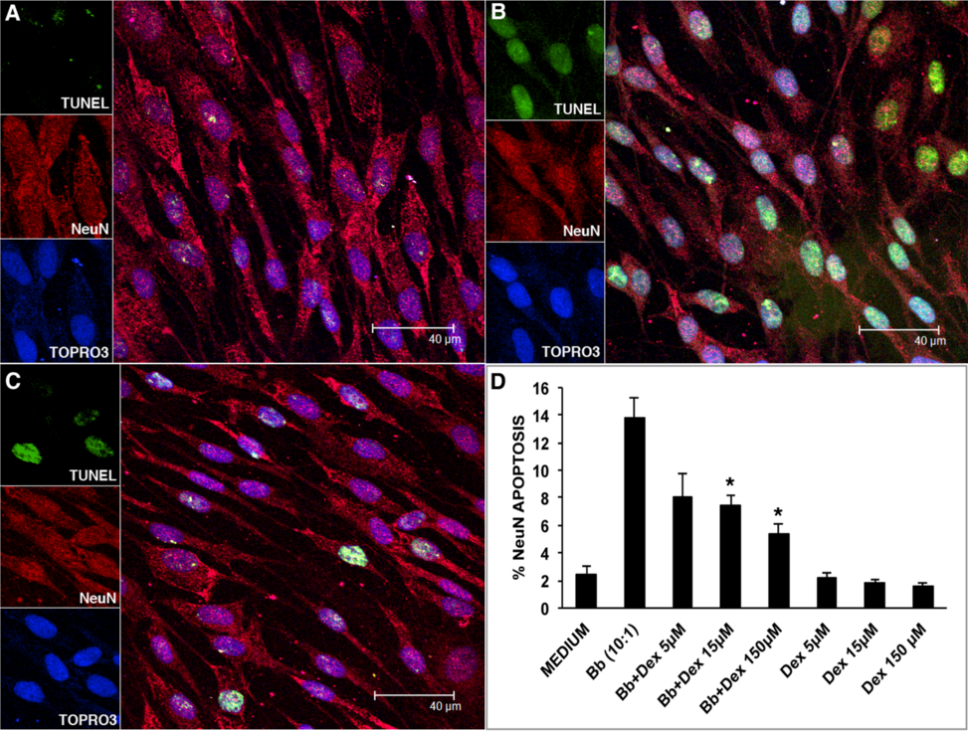
**Protective effect of dexamethasone on apoptosis induced by *B*. *burgdorferi *in primary rhesus DRG cultures. (A**-**C)** show representative images of sensory neurons in DRG cultures (red) staining with the neuronal marker NeuN, exposed to medium **(A)**, live *B*. *burgdorferi* at a MOI of 10:1 **(B)** and *B*. *burgdorferi* plus dexamethasone 15 μM **(C)**, respectively after 24 hours of incubation. Apoptotic nuclei are visualized in green as detected by the *in situ* TUNEL assay. The nuclei of all cells appear blue due to staining with the nuclear stain TOPRO3. **(D)** shows percent apoptosis as measured by the *in situ* TUNEL assay and quantified by confocal microscopy after 24 hours of incubation of DRG cultures exposed to medium or *B*. *burgdorferi* at a MOI of 10:1 in the presence and absence of dexamethasone (* *P* <0.05). Data represent percent apoptosis mean values from DRG cells that were isolated from two adult rhesus macaques. Values for each animal were determined in duplicate. Error bars represent standard deviations. DRG, dorsal root ganglia; MOI, multiplicity of infection; TUNEL, terminal deoxynucleotidyl transferase mediated UTP nick end labeling.

Live *B*. *burgdorferi* induced elevated levels of apoptosis of sensory neurons, as detected by the *in situ* TUNEL assay in primary rhesus DRG cultures, after 24 hours of incubation. Apoptosis visualized by confocal microscopy in cells that were held in medium alone is shown in Figure 5A, after incubation with live *B*. *burgdorferi* at MOI of 10:1 in Figure 5B and with *B*. *burgdorferi* plus dexamethasone at 15 μM in Figure 5C, respectively. The sensory neurons appear red due to staining with the neuronal marker NeuN. The TUNEL signal can be visualized in green while the nuclei of all cells appear blue due to staining with the nuclear stain TOPRO3. Figure 5D shows percent apoptosis as measured by the *in situ* TUNEL assay after 24 hours of incubation of DRG cultures in medium (2.41% ± 0.62), *B*. *burgdorferi* alone at a MOI of 10:1 (13.82% ± 1.39) and *B*. *burgdorferi* + dexamethasone at 5 μM, 15 μM and 150 μM in medium. Dexamethasone induced a significant reduction in *B*. *burgdorferi*-dependent apoptosis at 15 μM (7.39 ± 0.74) and 150 μM (5.32 ± 0.84) (*P* <0.05), respectively. The mean percent apoptosis and standard deviations quantified from ten microscope fields (a total of 500 cells) for each condition is shown in Figure 5D. The results represent the mean and standard deviation of values obtained from two independent experiments. No apoptosis was seen in the satellite glial cells in the primary rhesus DRG cultures, (not shown).

### Pro-inflammatory response induced by *B*. *burgdorferi* in HSC cultures

**Figure 6:**
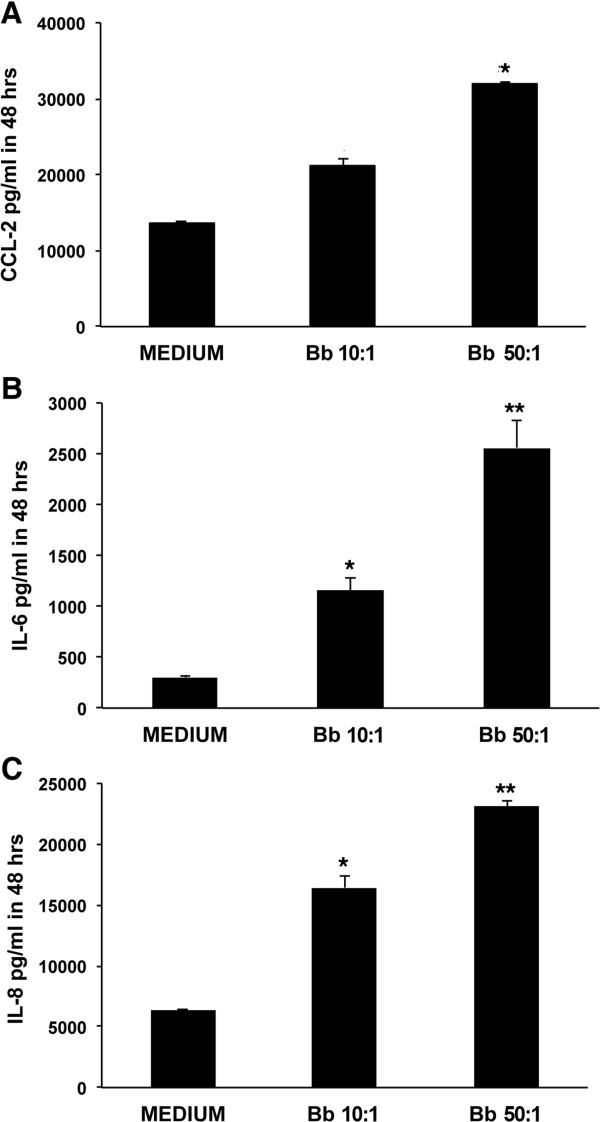
**Pro**-**inflammatory response induced by *B***. ***burgdorferi *in HSC cultures.** The levels of immune mediators CCL2 **(A)**, IL-6 **(B)** and IL-8 **(C)** in HSC cultures exposed to live *B*. *burgdorferi* spirochetes for 48 hours at a MOI of 10:1 and 50:1, respectively, and in medium controls are shown (* *P* <0.05, ** *P* <0.01). Data represent mean values and standard deviations between values of duplicate wells that were seeded for each condition for each of the two experiments set up using separate passage stocks. HSC, human Schwann cells; MOI, multiplicity of infection.

As we had detected the presence of CCL2, IL-6 and IL-8 in the dorsal roots of the DRG explants stimulated *ex vivo* with live *B*. *burgdorferi*, we evaluated the potential of live spirochetes to induce inflammatory mediators in *in vitro* cultures of pure HSC. Live *B*. *burgdorferi* spirochetes incubated with HSC cultures for 48 hours at a MOI of 10:1 and 50:1 induced significantly elevated levels of CCL2 (Figure 6A), IL-6 (Figure 6B) and IL-8 (Figure 6C) as compared to the levels induced in medium controls. The concentration of CCL2 surpassed 21,000 pg/mL and 32,000 pg/mL at a MOI of 10:1 and 50:1, respectively, whereas the constitutive level of this chemokine was about 13,000 pg/mL (Figure 6A). The basal concentration of IL-6 was only about 300 pg/mL but reached more than 1,150 pg/mL and 2,500 pg/mL at a MOI of 10:1 and 50:1, respectively (Figure 6B). Similarly, the basal concentration of IL-8 that was around 6,400 pg/mL reached more than 16,000 pg/mL and 23,000 pg/mL at a MOI of 10:1 and 50:1, respectively (Figure 6C). Data represent mean values and standard deviations between values of two independent experiments.

## Discussion

The pathogenesis of Lyme disease neuropathies is poorly understood. *B*. *burgdorferi* infection may damage neural cells by the direct action of spirochetes or spirochetal products on glial and neuronal cells. It is also possible that spirochetes induce cytotoxic or inflammatory mediators locally in glial, neuronal or endothelial cells and, thus, cause indirect damage. Infiltrating immune cells and/or the presence of cross-reactive antibodies to self-antigens at the site of infection/inflammation may also be deleterious to neural cells.

Neuronal proteins, anti-myelin antibodies and cells secreting antibodies to MBP have been detected in the CSF of patients with LNB, indicating possible glial and neuronal damage [32,33]. The antigenic determinants on the 41 kDa flagellar protein of *B*. *burgdorferi* are shared by several human tissue components such as Schwann cells from the PNS [34]. Long-term murine intrathecal exposure to a lipoprotein of *B*. *burgdorferi*, outer-surface protein C, resulted in axonal damage. Intrathecal exposure to *B*. *burgdorferi* lipoproteins may be one of the causes of the neurologic manifestations of Lyme disease [35]. The involvement of sensory ganglia in human LNB has also been previously documented [15,36]. One rare peripheral nervous system manifestation of LNB is the Guillain-Barré-like syndrome, a prototype of immune-mediated peripheral neuropathies where neither the initial event nor the antigen that triggers the immune reaction is known. Autoimmune mechanisms similar to those suggested in MS have also been implied in the pathogenesis of central nervous system LNB [37].

Our earlier findings in the rhesus model of peripheral LNB of both the early disseminated and chronic phases in the PNS mirror several aspects of these forms of the disease in humans [38,39]. The primary findings of axonal degeneration and regeneration, and multifocal nerve lesions showing perivascular inflammatory cellular infiltrates have been documented in almost all patients with Lyme-associated peripheral neuropathy [15,16,40-42]. The results of these studies suggest that immune mediated neuronal and glial cell damage could be involved in the neuropathy of LNB.

Cytokines and chemokines are key immune mediators that play an important role in promoting CNS injury in various kinds of inflammatory neurodegenerative diseases [43-47]. Importantly, various inflammatory cytokines and chemokines have also been reported in the CSF of patients with LNB [48-51]. The potential of *B*. *burgdorferi* to induce cytokines, chemokines and other inflammatory mediators in glial and neuronal cells as well as glial and neuronal apoptosis has been well documented [20,23,25,52-56].

In this study we explored the potential of the Lyme disease bacteria to cause inflammation in tissue explants of rhesus DRG, as well as in primary cultures of rhesus DRG cells and human Schwann cells, as a representation of the Schwann cells that ensheath the dorsal roots. Primary cultures of DRG cells from rhesus monkeys may prove useful in understanding the mechanisms involved in Lyme peripheral neuropathy, as well as in other human peripheral neuropathies [57].

We documented the ability of *B*. *burgdorferi* to elicit the production of IL-6, IL-8, and CCL2 in cells of the DRG and to induce the death of sensory neurons in DRG cell cultures. The ability of sensory neurons of the DRG to express CCL2 has also been documented in pain models [58]. Our *in vitro* DRG culture did not favor the growth of Schwann cells, which are known to ensheath the dorsal root axons in the DRG, possibly because the culture medium was tailored to supporting the neurons. Interestingly, the few satellite glial cells that were present in our DRG cultures did not undergo apoptosis in response to *B*. *burgdorferi*. We had previously observed the apoptosis of satellite glial cells in DRG of rhesus monkeys infected intrathecally with live *B*. *burgdorferi*[20]. It is possible that there are additional regulatory factors that come into play in the *in vivo* environment that are absent in the *in vitro* culture system. Specifically, this difference may have been brought about by the absence of Schwann cells in the DRG cultures, especially considering their documented contribution to inflammation in response to *B*. *burgdorferi*.

Using rhesus DRG tissue explants we identified the phenotypes of the producer cells as satellite glial cells by the expression of glial markers such as S-100 [59,60] and GFAP. Like other glial cells, the satellite glial cells are known to respond to nerve injury by up-regulating GFAP [61]. We identified Schwann cells in rhesus DRG tissue explants by the expression of p75NTR and CNPase [29,30,62]. These cells, in addition to DRG satellite cells, produced IL-6, IL-8 and CCL2, while neurons, which were characterized by the expression of NeuN [63], produced IL-6 and CCL2 but not IL-8. The DRG cell cultures as well as the cultures of HSC stimulated with live *B*. *burgdorferi* produced IL-6, IL-8 and CCL2. Since we did not find IL-8 to be produced by neurons in rhesus DRG explants, it is likely that satellite glial cells contributed the IL-8 found in the DRG cell culture supernatants.

These results support our hypothesis and show that innate responses of neuronal and glial cells of the DRG to *B*. *burgdorferi* mediate inflammation and that neuronal apoptosis occurs in this context. In agreement with this notion we found that the anti-inflammatory drug dexamethasone reduced both the levels of inflammatory mediators and neuronal apoptosis as induced by *B*. *burgdorferi*, suggesting that the two phenomena may be causally related.

Cytokine/chemokine signaling and apoptosis are of key importance in the regulation of neuroinflammatory responses [43-47]. Since DRG axons project centrally into the spinal cord and peripherally into the spinal nerves, inflammation and cell death in the DRG elicited by the Lyme disease spirochete could affect neuronal survival and function both in the CNS and PNS. Further, as the sensory neurons of the DRG play a key role in the sensation of pain, inflammation in glial and neuronal cells and cell death in the DRG could also modulate the pain response [64]. Neurogenic pain secondary to radiculitis or inflammation of the dorsal roots is often the earliest and sometimes only symptom in patients with LNB [65]. It typically radiates from the spine into the extremities or trunk, and is described as ‘sharp or jabbing’ pain [16]. The immune mediators IL-6, IL-8 and CCL2 that we found to be elevated in the DRG cultures exposed to live *B*. *burgdorferi* have been reported to play a role in modulating inflammation and the pain response [66-69]. IL-8 is known to induce expression of matrix metalloproteinases, cell cycle and pro-apoptotic proteins, and cell death in neurons [70]. IL-6 and CCL2 have been reported to increase the sensitivity of sensory neurons to pain [71-73].

The expression of the chemokine CCL2 and its receptor (CCR2) is also up-regulated by DRG neurons in rodent models of neuropathic pain [74]. Disruption of CCL2 signaling has been shown to block the development of neuropathic pain [75]. CCL2 is also known to be involved in the signaling and upregulation of several genes and proteins that participate in the signal transduction of the pain response both in the DRG and in the spinal cord [72,73]. This chemokine also functions as a neuromodulator in DRG neurons [74]. We observed the levels of CCL2 to be the highest among the immune mediators elicited by *B*. *burgdorferi* in both DRG cell and HSC cultures. Similarly, we have reported high levels of CCL2 in the CSF of rhesus macaques infected with *B*. *burgdorferi*[20]. It is possible that CCL2 is a major player in orchestrating inflammation as well as the pain response in LNB.

The local application of IL-6 to the DRG of rats has been shown to induce TNF-α and results in apoptosis of DRG cells [76]. Earlier, we also reported the presence of IL-6 in the sensory neurons of the DRG in rhesus macaques that were inoculated intrathecally with *B*. *burgdorferi*[20]. Patients with LNB sometimes have persistent symptoms such as fatigue, cognitive difficulties, depression and pain even after appropriate antibiotic treatment. Because of the subjective nature of these complaints, many of these patients are believed to have a primary psychiatric diagnosis, such as depression or somatization disorder. However, experiments such as those presented here raise the possibility that some of these complaints may be associated with inflammatory biochemical changes in the CNS. Elevated levels of IL-6 can cause symptoms of fatigue and malaise, common to many infectious conditions, as well as Lyme disease [77]. Research in other animal models of peripheral neuropathy has demonstrated that peripheral inflammation alone can trigger the brain cytokine system via afferent neural pathways from the periphery to the CNS [78]. Similar cascades of inflammation and apoptosis could also be involved in the DRG in LNB. Ongoing cytokine activation in the nervous system could contribute to the persistent symptoms of fatigue, pain and cognitive dysfunction that patients sometimes continue to experience despite having been treated for Lyme disease.

It is possible that *B*. *burgdorferi*, as well as the mediators elicited in cells of the DRG and Schwann cells that we report here, could contribute to mediating inflammatory and apoptotic signaling cascades in Schwann cells, the myelinating cells of the PNS. This, in turn, may result in axonal degeneration [79].

The potential of Schwann cells to initiate the process of Wallerian degeneration by releasing pro-inflammatory cytokines that are involved in leukocyte recruitment and differentiation (for example, IL-1β, CCL2, IL-8 and IL-6) has been documented [80]. As we found the inflammatory mediators IL-6, IL-8 and CCL2 in our HSC culture supernatants stimulated with live *B*. *burgdorferi*, as well as in the Schwann cells in the dorsal roots of the DRG tissue explants incubated with live *B*. *burgdorferi*, it is possible that similar mechanisms of inflammatory mediated axonal damage could be contributing to the peripheral neuritis seen in LNB.

The high levels of CCL2 that we found to be elicited in DRG neurons and satellite glial cells, as well as in Schwann cells in response to *B*. *burgdorferi*, could trigger mechanisms of demyelination in the PNS similar to those thought to cause CNS demyelination in MS and experimental autoimmune encephalomyelitis (EAE) [81,82]. CCR2, the receptor of CCL2, has been documented to be involved in macrophage recruitment to the injured PNS [83].

The ability of *B*. *burgdorferi* to activate the innate defense mechanisms of the host [84] and particularly in cells of the CNS is well documented [48,51,85-88]. It is possible that similar signaling cascades could be involved in triggering the immune mechanisms that result in the pathogenesis of peripheral LNB. We propose that inflammation of nerve roots and DRG and subsequent apoptosis in the DRG could be early events that contribute to peripheral neuropathy in Lyme neuroborreliosis.

## Conclusions

In this model, *B*. *burgdorferi* induced an inflammatory response and neuronal apoptosis of DRG. These pathophysiological processes could contribute to peripheral neuropathy in LNB.

## Abbreviations

CCL2: Chemokine (C-C) motif ligand 2; CCR2: C-C chemokine receptor 2; CD90: Cluster of differentiation 90; CNPase: 2′, 3′- cyclic nucleotide 3′-phosphodiesterase; CNS: Central nervous system; CSF: Cerebrospinal fluid; DIC: Differential interference contrast; DRG: Dorsal root ganglia; FBS: Fetal bovine serum; FSG: Fish skin gelatin; G-CSF: Granulocyte colony stimulating factor; GFAP: Glial fibrillary acidic protein; GM-CSF: Granulocyte macrophage colony stimulating factor; HPC: 2- hydroxypropyl β- cyclodextrin; HSC: Human Schwann cells; IFN-γ: Interferon gamma; IL-1ra: Interleukin 1 receptor antagonist; IL: Interleukin; LNB: Lyme neuroborreliosis; MBP: Myelin basic protein; MCP: Monocyte chemotactic protein; MOI: Multiplicity of infection; MS: Multiple sclerosis; NeuN: Nneuronal nuclear protein N; NGF: Nerve growth factor; NGS: Normal goat serum; P/S: Penicillin and streptomycin; p75NTR: Neurotrophin receptor p75; PBS-FT: Phosphate buffered saline fish skin gelatin Triton-X-100; PBS: Phosphate buffered saline; PFA: Paraformaldehyde; PNS: Peripheral nervous system; sCD40L: Soluble cluster of differentiation 40 ligand; SCM: Schwann cell medium; TGF-α: Transforming growth factor alpha; TNF-α: Tumor necrosis factor alpha; TUNEL: Terminal deoxynucleotidyl transferase mediated UTP nick end labeling; VEGF: Vascular endothelial growth factor

## Competing interests

The authors declare that they have no competing interests.

## Authors’ contributions

GR participated in the design of the experiments, conducted cell culture of DRG and HSC, *ex vivo* DRG tissue explant experiments, multiplex ELISA data analysis, immunofluorescence staining and confocal microscopy and drafted the manuscript. LSG helped in cell culture, immunofluorescence staining and multiplex ELISA data analysis of HSC. FI helped in establishing primary DRG cell cultures. JDE contributed to editing the manuscript. MTP conceived of the study, contributed to the design of the experiments, and to drafting and editing the manuscript. All authors have read and approved the final version of the manuscript.
